# Efficacy of thoracoscopic segmentectomy versus lobectomy in the treatment of early invasive lung adenocarcinoma: a propensity score matching study

**DOI:** 10.3389/fonc.2023.1186991

**Published:** 2023-08-31

**Authors:** Congyi Ding, Qiyu Jia, Zhongjie Wu, Yanfei Zhang, Yi Hu, Jingyu Wang, Dahai Wei

**Affiliations:** ^1^ Jiaxing University Master Degree Cultivation Base, Zhejiang Chinese Medical University, Jiaxing, Zhejiang, China; ^2^ Department of Trauma Orthopedics, The First Affiliated Hospital of Xinjiang Medical University, Urumqi, Xinjiang, China; ^3^ Department of Cardiothoracic Surgery, The First Hospital of Jiaxing, Jiaxing, Zhejiang, China

**Keywords:** invasive adenocarcinoma of lung, segmentectomy, lobectomy, immunoinflammatory response, retrospective analysis

## Abstract

**Objective:**

This study aimed to investigate and analyze the clinical application value of thoracoscopic segmentectomy and lobectomy in patients with invasive pulmonary adenocarcinoma.

**Methods:**

286 patients with invasive pulmonary adenocarcinoma who underwent segmentectomy or lobectomy at the First Hospital of Jiaxing City from January 2018 to June 2020 were retrospectively analyzed. Patients were divided into a thoracoscopic segmentectomy group(n=97) and a lobectomy group (n=189). Patients were compared after obtaining 1:1 propensity score-matched cohorts. Outcome indicators included surgery-related indicators, immune-inflammation-related indicators, postoperative complications, recurrence, and metastasis.

**Results:**

After 1:1 propensity score matching, 93 patients were included in each group. We found that the volume of intraoperative blood loss in the segmentectomy group was significantly less than in the lobectomy group (P=0.014). The duration of postoperative drainage (P = 0.005) and hospitalization (P=0.002) in the segmentectomy group were significantly shorter than in the lobectomy group. In terms of immunoinflammatory response, compared with the lobectomy group, white blood cells, neutrophils, SII, and NLR in the segmentectomy group were significantly lower than in the lobectomy group (P< 0.05). The recurrence-free survival (RFS) rates in the segmentectomy and lobectomy were 80.5% and 88.2% at 1 year and 35.1% and 52.6% at 3 years, respectively, and the difference was statistically significant (P<0.05). The segmentectomy group achieved similar outcomes to the lobectomy group at 1 year and 3 years (P > 0.05). Multivariate COX regression analysis showed that CAR was an independent risk factor for RFS in patients undergoing invasive adenocarcinoma surgery.

**Conclusion:**

Compared with lobectomy, thoracoscopic segmentectomy can effectively reduce the postoperative inflammatory response in patients with early invasive lung adenocarcinoma and promote patient recovery. Although segmentectomy is associated with a higher recurrence rate in the short term for patients with early invasive lung adenocarcinoma, the associated survival rate is similar to the lobectomy group. Segmentectomy should be considered in the treatment of early invasive lung adenocarcinoma. Meanwhile, postoperative CAR represents an independent risk factor for early postoperative recurrence in patients with IAC.

## 1 Introduction

Lung cancer is the leading cause of cancer-related mortality worldwide (1). Non-small cell lung cancer (NSCLC) is the most common subtype of lung cancer, accounting for 84% of new cases (2). Lobectomy remains the mainstay of treatment for early-stage lung cancer (3, 4). However, it should be borne in mind that different procedures can cause different severities of trauma to the body, leading to varying levels of inflammation. This inflammatory reaction is present from the beginning of the operation and continues throughout the postoperative recovery process, affecting the recovery time. Surgeons have long sought to minimize surgical trauma. Currently used systemic inflammatory response-based scores (5) during clinical practice include the Systemic Immunoinflammatory Index (SII) (6), Neutrophil/Lymphocyte Ratio (NLR) (7), Platelet/Lymphocyte Ratio (PLR), C-reactive protein/albumin ratio (CAR) (8) and Prognostic Nutritional Index (PNI) (9).

In 2011, the World Health Organization (WHO) classified lung epithelial tumors into preinvasive and invasive lesions (10). The pre-infiltrative lesions typically include atypical adenomatous hyperplasia (AAH) and adenocarcinoma in situ (AIS), and invasive lesions include microinvasive adenocarcinoma (MIA) and invasive adenocarcinoma (IAC). In 2021, the WHO proposed a new classification of lung adenocarcinoma (11), classifying epithelial lung tumors as benign, precursor lesions, and adenocarcinoma, including MIA and IAC. IAC is a malignant epithelial tumor with adenoidal differentiation that produces mucus or expresses alveolar cell markers. Pathologically, this tumor type infiltrates the surrounding tissue containing myofibroblasts forming vesicular, papillary, micropapillary, and solid growth patterns exceeding 5 mm in size. Regardless of the tumor size and extent of infiltration, tumor cells invade the vasculature or extend into the pleura, often accompanied by necrosis and spread into airspaces. IAC consists of five subtypes (12): adherent growth, acicular, papillary, solid, and micropapillary, with the solid and micropapillary types being the least differentiated and most malignant. Indeed, it is well-established that the malignancy of the tumor directly affects the patient’s outcome (13). Patients with pathological evidence of AIS or MIA experience an extremely high survival rate after resection. Little is currently known about the optimal surgical approach to early-stage lung cancer (14), nor is it unclear whether the clinical efficacy of segmentectomy is equivalent to lobectomy in patients with early-stage IAC pathology, given that the postoperative inflammatory response to both has been rarely explored.

In this study, we retrospectively analyzed the clinical data of patients who underwent thoracoscopic segmentectomy or lobectomy for early-stage IAC at Jiaxing First Hospital and evaluated the perioperative and early outcomes of segmentectomy versus lobectomy by assessing patients’ surgery-related data, immunoinflammatory response-related data, 3-year OS and RFS, which may provide the foothold for future studies on the advantages of segmentectomy over lobectomy for early-stage IAC patients.

## 2 Materials and methods

### 2.1 Patient and method

We included 286 patients who underwent surgical treatment in the Department of Thoracic Surgery at Jiaxing First Hospital from January 2018 to June 2020 with pathological evidence of IAC. These patients were divided into a segmentectomy group [n=97, female (n=63, 64.9%), median age 63 (Range 54~69) years] and a lobectomy group [n=189, female (n=121, 64.0%), median age 63 (55~68.5) years]. The inclusion criteria were as follows: segmentectomy or lobectomy; IA patients with stage I pathology, tumor diameter ≤ 3 cm (staging T1a-1cN0M0), the distance between cut edges is greater than tumor diameter; no preoperative antitumor treatment such as radiotherapy, chemotherapy, immunotherapy or targeted therapy; complete clinical data. The exclusion criteria consisted of patients that underwent previous radiotherapy, chemotherapy, immunotherapy, or targeted therapy; open thoracic surgery or thoracoscopic wedge resection of the lung; non-invasive adenocarcinoma of the lung on pathology, stage II or higher invasive adenocarcinoma of the lung, malignant neoplasm of the lung other than adenocarcinoma, metastatic malignant neoplasm of the lung; incomplete clinical data. The study was approved by the ethical review committee of Jiaxing First Hospital (ethical approval number:2022-LY-399), and all patients provided informed consent.

### 2.2 Preoperative evaluation

All patients were examined before admission, including complete blood count, preoperative infectious serological tests (HIV-ab, HCV-ab, Hbs-ag, tp-ab), biochemical analysis, electrocardiogram, brain CT, chest CT scan, abdominal ultrasound, vascular ultrasound, cardiac ultrasound, superficial lymph node ultrasound, and pulmonary function. Some patients underwent whole-body bone scans, brain MR and bronchoscopy. Tumors were classified based on their imaging presentation as pure ground-glass opacity (pGGO), mixed ground-glass opacity (mGGO), and solid tumors (Solid).

### 2.3 Observation indexes

(1) Preoperative general information: gender, age, body mass index (BMI), smoking history, and preoperative comorbidities; (2) Operative data: time of operation, intraoperative bleeding, number of intraoperative lymph node dissection groups, postoperative day 1 drainage, total postoperative drainage within 3 days, time of drainage tube removal, postoperative hospital days; (3) Clinicopathological and imaging data: including pathological stage, degree of tumor differentiation, tumor diameter, tumor imaging performance; (4) Systemic inflammation-related laboratory markers: including postoperative white blood cells (WBC), lymphocytes (LC), neutrophils (NE), platelets (PLT), albumin (ALB), etc., calculation of SII, NLR, PLR, CAR, PNI. All results were obtained within three days of the operation. (5) Postoperative complication data: postoperative pulmonary atelectasis, postoperative pulmonary infection, persistent postoperative pulmonary air leak, and postoperative lower limb venous thrombosis; (6) Survival data: 3-year OS (overall survival) and 3-year RFS (recurrence-free survival). Calculation formula: SII=(PLT×NE)/LC; NLR=NE/LC; PLR=PLT/LC; CAR=CRP/ALB; PNI=ALB+5×LC;

### 2.4 Surgical methods

In the observation group, patients underwent thoracoscopic segmentectomy. After induction of general anesthesia with the patient in the decubitus position, the one-port or two-port method was utilized. A thoracoscope was inserted through the main port, and a wedge-shaped lung resection was performed. The resected tissue samples were sent for a rapid frozen section examination during the operation. The dissection began at the root of the lung segment and progressed in the same direction, gradually exposing the superficial segmental blood vessels and segmental bronchi in the surgical field and dissecting the connection between the lung segments. Prior to clipping the segmental bronchus, the lung was expanded with the assistance of an anesthesiologist to ensure that the segmental bronchus to be clipped was the target segmental bronchus. During the operation, the surgeon used a thoracoscopic incision stapler to resect the intersegmental fissure during the operation. The surgeon ensured a safe margin of at least 2cm and excised it to guarantee complete removal. After the excision, the specimen was verified to ensure its adequacy. During the operation, lymph node sampling was simultaneously performed and sent for frozen section examination. A lobectomy was performed if the pathological results indicated the presence of lymph node metastasis. Additionally, intraoperative frozen pathology was used to assess the distance of the incision margin to ensure an appropriate margin was achieved during the surgical procedure.

In the control group, thoracoscopic lobectomy was performed on the patients. The operating position, anesthesia method, and operation ports were similar to the observation group. The chemical glue was injected next to the tumor under the guidance of CT before the operation to locate the tumor. During the operation, the resected nodules were sent for rapid frozen section examination. Using linear cutting and a stapler, the bronchus, pulmonary vein, pulmonary artery, and dysplastic interlobar fissure were severed, and the lobe containing the lesion was resected.

### 2.5 Statistical analysis

SPSS 23.0 was used for statistical analysis, and GraphPad Prism was used to generate graphs. Normally distributed data were expressed as (x ± s), and the t-test for independent samples was used to compare two groups; non-parametric data were expressed as Median (P25, P75), and the non-parametric test was used to compare groups. Recurrence-free survival (RFS) was defined as the time from surgery to recurrence, death from any cause, or last follow-up date. Overall survival (OS) was the time from surgery to death from any cause or last follow-up date. Survival data were calculated using the Kaplan-Meier method and compared using the log-rank test. With RFS as the outcome variable, Kaplan-Meier curves were used to generate receiver operating characteristic (ROC) curves for each indicator of the postoperative immune response, and optimal cutoff values were derived from the maximum Youden index. Each indicator of the postoperative immune inflammatory response was stratified into high and low groups according to the optimal cutoff value. Cox proportional regression analysis was used to explore factors influencing the development of RFS in patients with invasive adenocarcinoma of the lung. Differences were considered statistically significant at P<0.05. To minimize the influence of variations in baseline data among patients undergoing thoracoscopic lung segmentation and lobectomy on the final results, the researchers employed propensity score matching (PSM) using the proximity matching method. This matching process aimed to pair patients from both groups in a 1:1 ratio based on their propensity scores. Variables used to construct the propensity score included: age, gender, BMI, smoking history, pulmonary infection, hypertension history, diabetes history, pathologic stage, cardiac arrhythmia, tumor size, and carcinoembryonic antigen. Propensity score matching (PSM) analysis was performed using STATA16, ensuring covariate balance. Subgroup analysis using STATA16 investigated the effects of lung segment therapy and lung lobe therapy within different pathological stages and imaging manifestations. The COX proportional risk model was used for analysis, and the final results were presented through a forest plot generated with R software.

### 2.6 Follow-up after surgery

The patients were followed up postoperatively, and serum tumor markers, HRCT of the lungs, and ultrasound of the abdomen were conducted every 6-12 months. When recurrence or metastasis was suspected, further evaluation was performed, including chest CT or PET-CT scan, whole body bone scan, and cranial MRI. Local recurrence was defined as occurring in the ipsilateral half, including the lungs, lymph nodes, and pleura, and distant recurrence was defined as distant organ metastases.

## 3 Results

### 3.1 Propensity score matching results

Finally, 186 patients were successfully matched in the segmentectomy [n=93, 31 males, 62 females, aged 63 (54-69) years] versus the lobectomy group [n=93, 30 males, 63 females, aged 61 (54-66)]. The differences in confounding variables between the two groups after matching were not significant (p>0.05) (**Tables 1**, **2**). A total of 286 participants were included in the study. Since the overall disease recurrence rate was unknown, a maximum difference was assumed with *pq*=0.5∗0.5 = 0.25. To ensure a sampling error limit of no more than 5% and achieve a 95% confidence level for random matching, the minimum required sample size is calculated as follows:

**Table 1:** General information before matching.

	Segmentectomy group (n=97)	Lobectomy group (n=189)	*Z*	*P*
Gender				0.877
Male	34(35.1%)	68(36%)		
Female	63(64.9%)	121(64%)		
Age [years, M (P25, P75)]	63(54~69)	63(55~68.5)	-0.041	0.967
BMI (Kg/m^2^)	23.66 ± 3.07	23.45 ± 2.98	-0.578	0.564
Size [cm, M (P25, P75)]	1.3(1.1~1.6)	1.4(1.2~2)	-1.893	0.058
Pathologic Stage			-0.59	0.611
T1 a	16(16.5%)	42(22.2%)		
T1 b	77(79.4%)	120(63.5%)		
T1 c	4(4.1%)	27(14.3%)		
Imaging Performance			-0.137	0.891
Pure	40(41.2%)	48(25.4%)		
Solid	17(17.5%)	90(47.6%)		
Mix	40(41.2%)	51(27%)		
Tumor Differentiation Grade			-0.875	0.172
Low-grade	8(8.2%)	22(11.6%)		
Intermediate-grade	80(82.5%)	152(80.4%)		
High-grade	9(9.3%)	15(7.9%)		
Smoking history	14(14.4%)	30(15.9%)		0.749
Preoperative Complications	40(41.2%)	98(51.9%)		0.089
Pulmonary Infection	9(9.3%)	18(9.5%)		0.946
Arrhythmia	10(10.3%)	10(5.3%)		0.115
Hypertension	30(30.9%)	79(41.8%)		0.073
Diabetes	9(9.3%)	21(11.1%)		0.632
Preoperative Carcinoembryonic antigen	2(1.3~3)	2.3(1.4~3.3)	-1.367	0.172

**Table 2:** General information after matching.

	Segmentectomy group (n=93)	lobectomy group (n=93)	*Z*	*P*
Gender				0.876
Male	31(33.3%)	30(32.3%)		
Female	62(66.7%)	63(67.7%)		
Age [years, M (P25, P75)]	63(54~69)	61(54~66)	-1.051	0.293
BMI (Kg/m^2^)	23.6 ± 3.03	23.6 ± 2.65	-0.042	0.967
Size [cm, M (P25, P75)]	1.3(1.1~1.6)	1.3(1.2~1.75)	-0.053	0.957
Pathologic Stage			-0.163	0.871
T1 a	16(17.2%)	21(22.6%)		
T1 b	73(78.5%)	61(65.6%)		
T1 c	4(4.3%)	11(11.8%)		
Imaging Performance			-0.08	0.937
Pure	38(40.9%)	28(30.1%)		
Solid	17(18.3%)	38(40.9%)		
Mixed	38(40.9%)	27(29%)		
Tumor Differentiation Grade			-0.362	0.717
Low-grade	8(8.6%)	6(6.5%)		
Intermediate-grade	77(82.8%)	79(84.9%)		
High-grade	8(8.6%)	8(8.6%)		
Smoking History	12(12.9%)	11(11.8%)		0.824
Preoperative complications	36(38.7%)	36(38.7%)		1
Pulmonary Infection	8(8.6%)	7(7.5%)		
Arrhythmia	6(6.5%)	6(6.5%)		
Hypertension	28(30.1%)	27(29%)		
Diabetes	8(8.6%)	9(9.7%)		
Preoperative Carcinoembryonic antigen	2(1.25~2.95)	2.2(1.25~3.1)	-0.128	0.898


$$n=\frac{NZ_{\alpha /2}^{\;2}pq}{N{\text{Δ}}_{p}^{\;2}+Z_{\alpha /2}^{\;2}pq}=\frac{286*1.96^{2}*0.5*0.5}{286*{\left(5\%\right)}^{2}+1.96^{2}*36.56\%*63.44\%}=171$$

The sample size of 186, obtained through 1:1 matching, satisfies the inspection requirements as outlined in this paper.

### 3.2 Propensity score balance test

**Figure 1:**
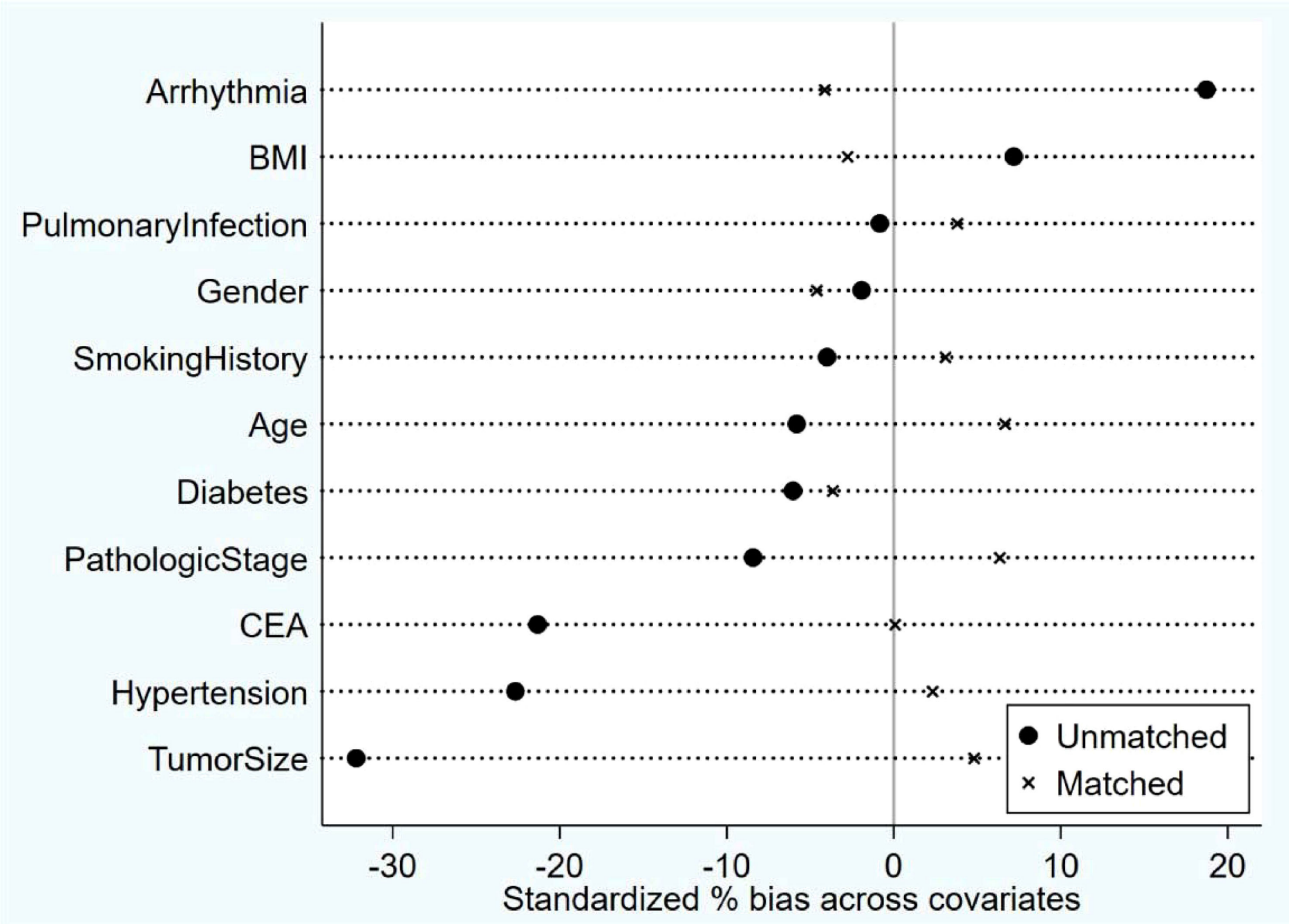
Post-Matching balance test results: After matching, all covariates demonstrated less than 10% standardization bias, with no significant differences observed. The Propensity Score Matching (PSM) method was used for matching.

The study conducted a propensity score balance test to assess the distribution of covariates before and after implementing the PSM method. The preferred PSM approach was K-nearest neighbor 1:1 without replacement, with a caliper set at 0.02. Before matching, some variables, such as arrhythmia, CEA, hypertension, and tumor size, showed substantial standardization bias exceeding 20%. However, after matching, all covariates demonstrated less than 10% standardization bias, with no significant differences observed. These results indicated that the propensity score matching was effective in achieving balance among the covariates. The balance test confirmed the success of the matching process (**Figure 1**).

### 3.3 Perioperative results

Intraoperative bleeding was significantly lower in the segmentectomy group than in the lobectomy group [(50(30-50)) ml vs. (50(50-80)) ml, P=0.014]. The duration of postoperative drainage was significantly less in the segmentectomy group than in the lobectomy group [(4(3-6)) days vs. (5(3.5-7)) days, P=0.005]. The postoperative hospitalization was significantly shorter in the segmentectomy group than in the lobectomy group [(6(5-8)) days vs. (7(6-10)) days, P=0.002]. However, there were no statistically significant differences (P>0.05) between the two groups in terms of the drainage volume on postoperative day 1, the total drainage volume within 3 days postoperatively, the number of lymph node dissection groups, and the occurrence of postoperative complications. 46.8% (87/186) of patients with invasive adenocarcinoma developed complications after surgery. Complications in the segmentectomy group included postoperative atelectasis (n=3, 3.2%), pulmonary infection (n=39, 41.9%), persistent air leakage (n=17, 18.3%), and lower extremity venous thrombosis (n=10, 10.8%). The corresponding complication rates in the lobectomy group were 3.2% (n=3), 39.8% (n=37), 15.1% (n=14), and 7.5% (n=7), respectively (**Table 3**).

**Table 3:** Perioperative results.

	Segmentectomy group (n=93)	Lobectomy group (n=93)	*Z*	*P*
Intraoperative Bleeding	50(30~50)	50(50~80)	-2.451	0.014
Drainage volume on the first day after operation	130(77.5~225)	120(65~212.5)	-0.634	0.526
Drainage volume 3 days after operation	510(345~702.5)	625(340~832.5)	-1.79	0.074
Drainage days	4(3~6)	5(3.5~7)	-2.824	0.005
Number of lymph node dissection	5(3~6)	5(3~6)	-0.539	0.59
Postoperative hospital stay	6(5~8)	7(6~10)	-3.031	0.002
Postoperative complication	46(49.5%)	41(44.1%)		0.462
Postoperative atelectasis	3(3.2%)	3(3.2%)		
Postoperative pneumonia	39(41.9%)	37(39.8%)		
Postoperative lung leakage	17(18.3%)	14(15.1%)		
Postoperative venous thrombosis of the lower extremities	10(10.8%)	7(7.5%)		

### 3.4 Systemic immune inflammatory response index results

Patients in the segmentectomy group exhibited significantly lower postoperative leukocytes than the lobectomy group [(9.82 ± 3.24)×10^9^/L vs. (11.28 ± 3.25) ×10^9^/L, P=0.02]. Postoperative neutrophils were lower in the segmentectomy group than in the lobectomy group [(8.32 ± 2.98) ×10^9^/L vs. (9.59 ± 2.95) ×10^9^/L, P=0.04]. Patients in the segmentectomy group had lower postoperative SII than the lobectomy group [1468.92 (888.695~2055.6) vs. 1633.36 (1169.715~2320.295), P=0.047]. Postoperative NLR was lower in patients in the segmentectomy group than in the lobectomy group [7.58 (5.64-11.4) vs. 9.08 (6.89-11.95), P=0.049]. There was no significant significance in postoperative lymphocytes, platelets, albumin, C-reactive protein, D-dimer, PLR, CAR, and PNI (P_s_>0.05) (**Table 4**).

**Table 4:** Results of immunoinflammatory indexes.

	Segmentectomy group (n=93)	Lobectomy group (n=93)	Z/t	P
Postoperative white blood cells	9.82 ± 3.24	11.28 ± 3.25	-3.07	0.02
Postoperative neutrophils	8.32 ± 2.98	9.59 ± 2.95	-2.92	0.04
Postoperative lymphocytes	1(0.8~1.3)	1.1(0.8~1.3)	-0.79	0.429
Postoperative platelets	182.44 ± 57.33	186.92 ± 54.84	-0.545	0.586
Postoperative albumin	36.14 ± 3.05	36.01 ± 3.23	-0.266	0.79
Postoperative CRP	29.3(18.1~42.85)	25.3(17.9~32.65)	-1.371	0.17
Postoperative D-dimer	1070(660~1855)	960(635~1845)	-0.695	0.487
Postoperative SII	1468.92(888.69~2055.6)	1633.36(1169.71~2320.29)	-1.99	0.047
Postoperative NLR	7.58(5.64~11.4)	9.08(6.89~11.95)	-1.967	0.049
Postoperative PLR	176.67(138.89~245)	178.33(132.985~231.965)	-0.019	0.985
Postoperative CAR	0.79(0.49~1.19)	0.69(0.5~0.935)	-1.284	0.199
Postoperative PNI	41.6(38.8~44.05)	41.8(38.9~44)	-0.463	0.643

### 3.5 Prognosis

**Figure 2:**
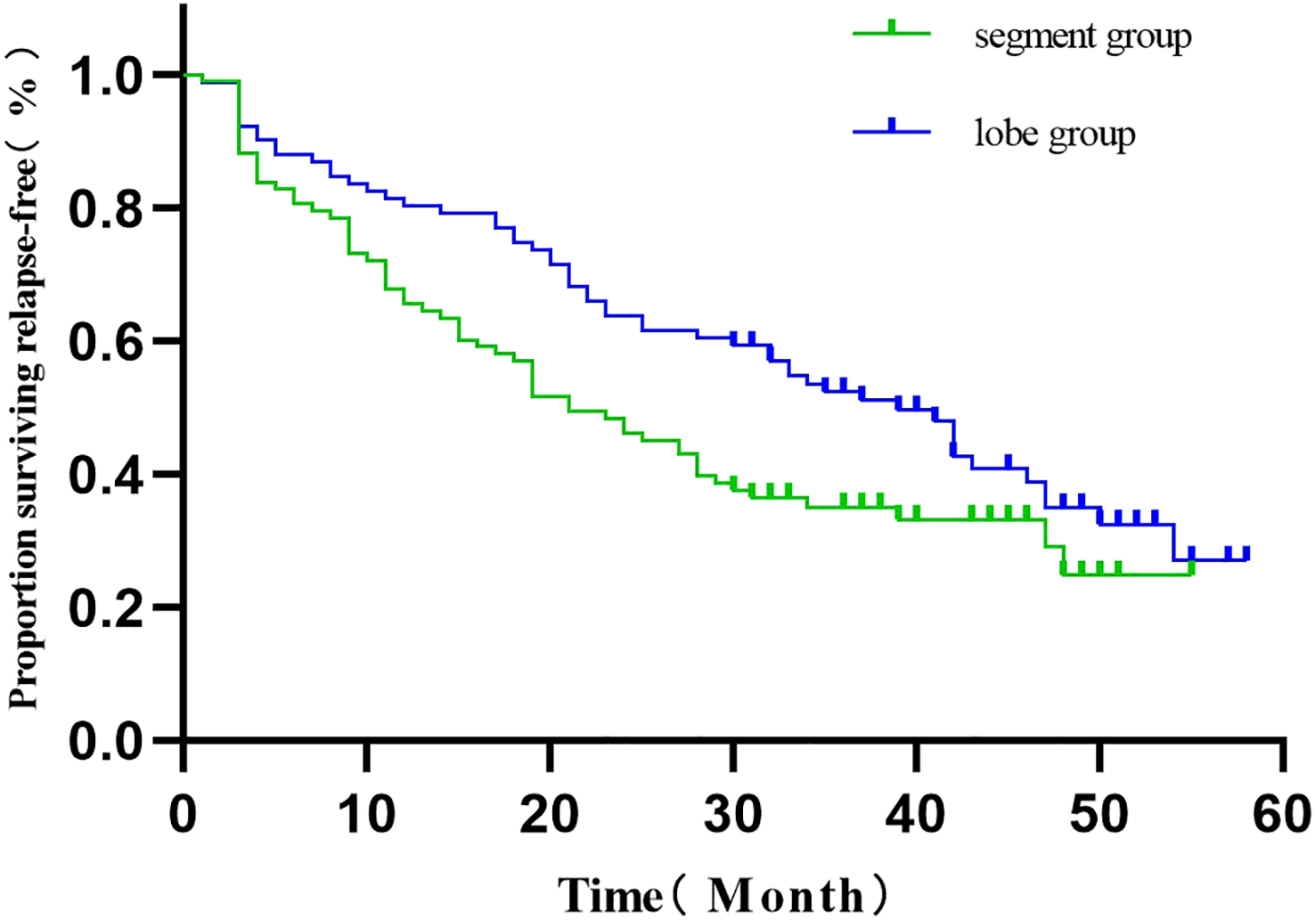
Comparison of RFS curves (Kaplan-Meier, Log-Rank test:P=0.029) showed statistically significant differences.

**Figure 3:**
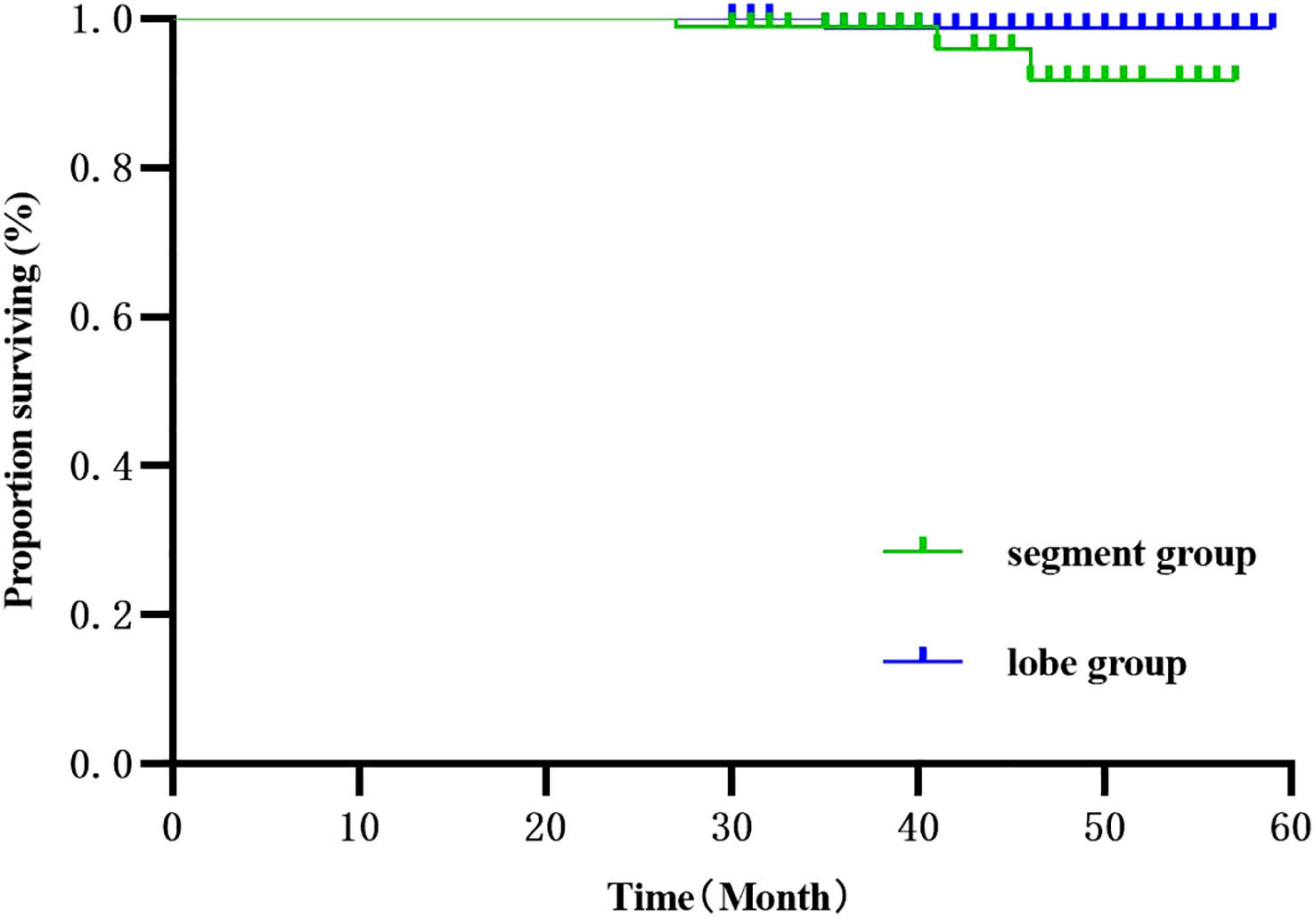
Survival curve comparison (Kaplan-Meier, Log-Rank test:P=0.161) showed no statistical significance.

The 186 patients were followed up for 30-59 months, with 4 deaths and 118 recurrences and metastases. Three deaths were observed in the segmentectomy group attributed to cancer (n=1) and non-cancer (n=2)-related deaths. Only one case of death was observed in the lobectomy group. The RFS of the segmentectomy and lobectomy was 80.5% vs. 88.2% at 1 year, and at 3 years, it was 35.1% vs. 52.6%, respectively. The hazard ratio for disease recurrence or death was 1.516, with a 95% CI of 1.045 to 2.201 during Kaplan-Meier survival analysis and a significant difference between the two groups (P=0.029) (**Figure 2**). The 1-year and 3-year postoperative OS in the segmentectomy and lobectomy groups were comparable (100% vs. 100% and 98.8% vs. 98.9%, respectively), and the hazard ratio for death, 0.221; 95% CI, 0.023 to 2.155 during Kaplan-Meier survival analysis, with no significant difference between the two groups (P=0.161) (**Figure 3**).

### 3.6 Postoperative NLR, PLR, CAR, SII, PNI optimal cutoff values

**Figure 4:**
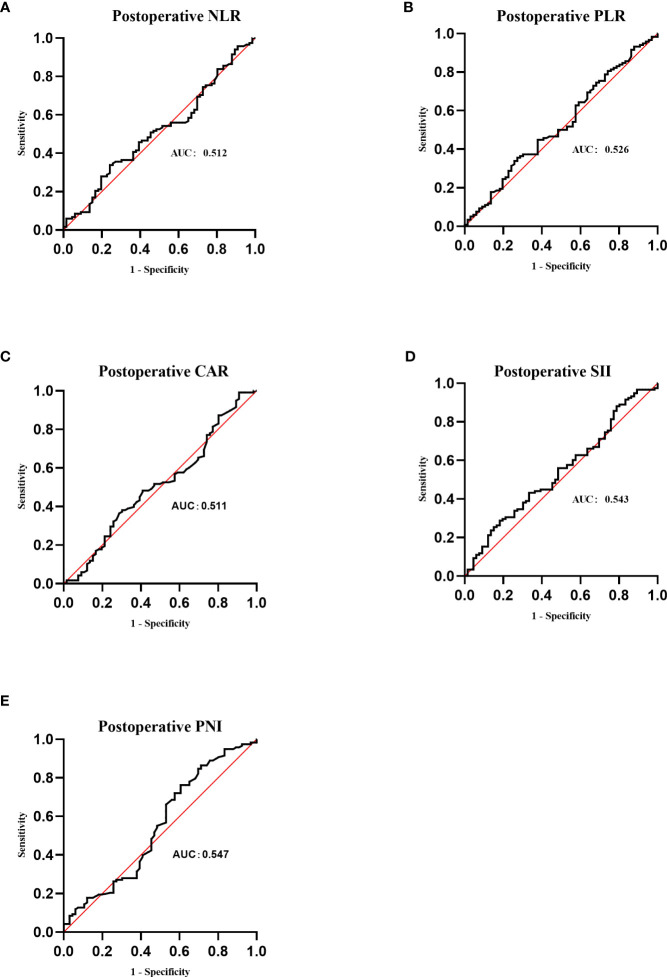
Receiver operating characteristic (ROC) curves of postoperative NLR, PLR, CAR, SII and PNI. **(A)** ROC curve analysis of postoperative NLR in patients with invasive lung adenocarcinoma. **(B)** ROC curve analysis of postoperative PLR in patients with invasive lung adenocarcinoma. **(C)** ROC curve analysis of postoperative CAR in patients with invasive lung adenocarcinoma. **(D)** ROC curve analysis of postoperative SII in patients with invasive lung adenocarcinoma. **(E)** ROC curve analysis of postoperative PNI in patients with invasive lung adenocarcinoma.

As shown in **Figure 4**, the postoperative area under the curve (AUC) was 0.512, 0.526, 0.511, 0.543, and 0.547 for NLR, PLR, CAR, SII, and PNI, respectively. The optimal postoperative cutoff values for NLR, PLR, CAR, SII, and PNI were 9.52, 181.29, 0.25, 5,876.70, and 39.65, respectively. We divided the patients into high and low groups for further analysis according to the optimal cutoff values. Of these, 110 (59.1%) patients had a postoperative NLR ≤9.52 and 76 (40.9%) patients had a postoperative NLR >9.52. The postoperative PLR was ≤181.29 in 98 (52.7%) patients and >181.29 in 88 (47.3%) patients. The postoperative CAR was ≤0.25 in 7 (3.8%) patients and >0.25 in 179 (96.2%) patients. 183(98.4%) patients had a postoperative SII ≤ 5876.70,3(1.6%) patients had a postoperative SII > 5 876.70. The postoperative PNI was ≤39.65 in 59 (31.7%) patients and >39.65 in 127 (68.3%) patients.

### 3.7 Univariate and multivariate COX regression analysis

**Figure 5:**
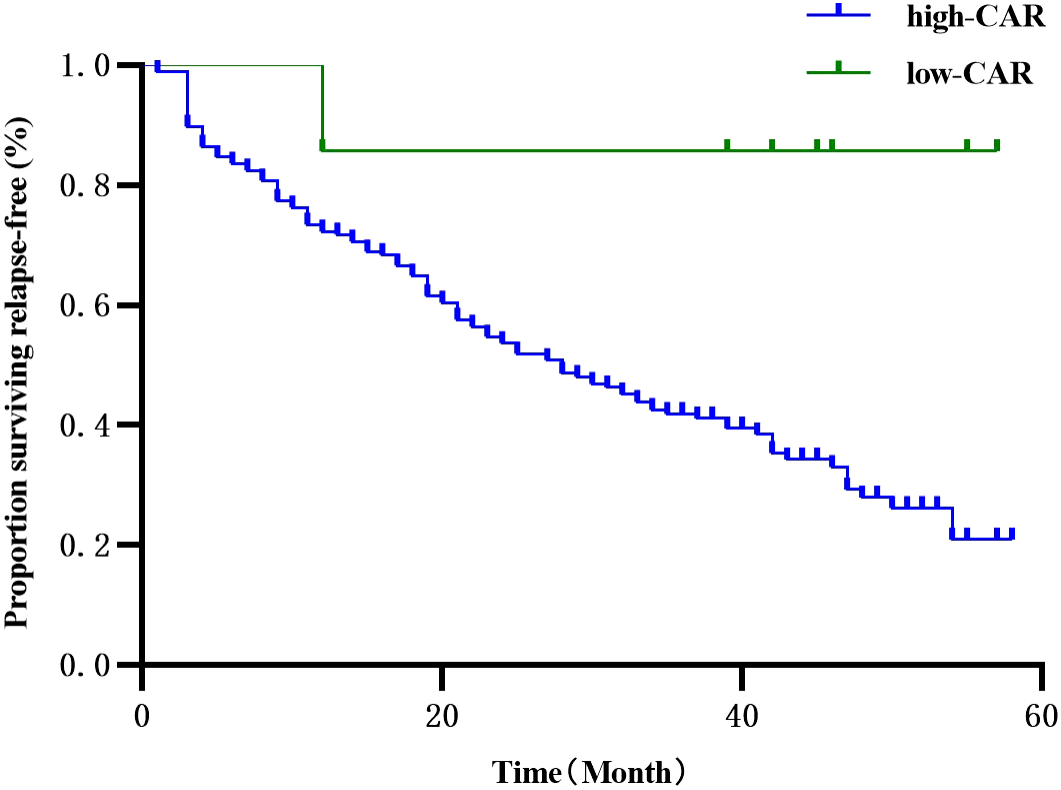
Univariate survival analysis of Kaplan-Meier curve describing the effect of CAR on the prognosis of patients with RFS after lung cancer resection.

Univariate COX regression analysis showed that the surgical approach, smoking history, tumor diameter, pathological stage, and postoperative CAR were associated with RFS. Age, gender, BMI, degree of tumor differentiation, imaging presentation, days of drainage, number of lymph node dissection groups, postoperative NLR, PLR, PNI, and SII were not associated with RFS (**Table 5**). Multi-variate COX regression analysis showed that postoperative CAR was an independent risk factor for RFS in patients undergoing IAC surgery, and CAR > 0.25 suggested a greater risk of recurrence (**Table 6**, **Figure 5**).

**Table 5:** Univariate analysis (n=186).

Variable	HR(95%CI)	P value
Surgical Approach	1.501 (1.042 -2.162)	0.029
Age	1.002 (0.984 -1.020)	0.865
Gender	0.816 (0.550-1.212)	0.314
BMI	0.983 (0.923-1.046)	0.583
Smoking history	0.546 (0.293-1.018)	0.039
Size	0.668 (0.446-1.000)	0.044
Pathological stage (T1 a-b, T1 c)	0.292 (0.108-0.794)	0.016
Tumor differentiation grade (low-grade, middle/high-grade)	0.827 (0.526-1.302)	0.412
Imaging performance (pure/solid, mix)	1.166 (0.801-1.699)	0.422
Postoperative NLR (≤9.52,>9.52)	1.804 (0.753-1.562)	0.664
Postoperative PLR (≤181.29,>181.29)	0.998 (0.695-1.432)	0.99
Postoperative CAR (≤0.250,>0.250)	7.346 (1.023-52.743)	0.047
Postoperative PNI (≤39.65,>39.65)	1.313 (0.875-1.970)	0.188
Postoperative SII (≤5876.70,>5876.70)	2.262 (0.714-7.170)	0.165

**Table 6:** Multivariate analysis.

Variable	HR (95%CI)	P value
Surgical Approach	1.400(0.971-2.020)	0.072
Smoking history	0.569(0.304-1.067)	0.079
Pathological stage (T1 a-b, T1 c)	0.376(0.119-1.187)	0.095
Size	0.892(0.543-1.463)	0.650
Postoperative CAR (≤0.250, >0.250)	8.507(1.181-61.274)	0.043

### 3.8 Subgroup analysis

**Figure 6:**
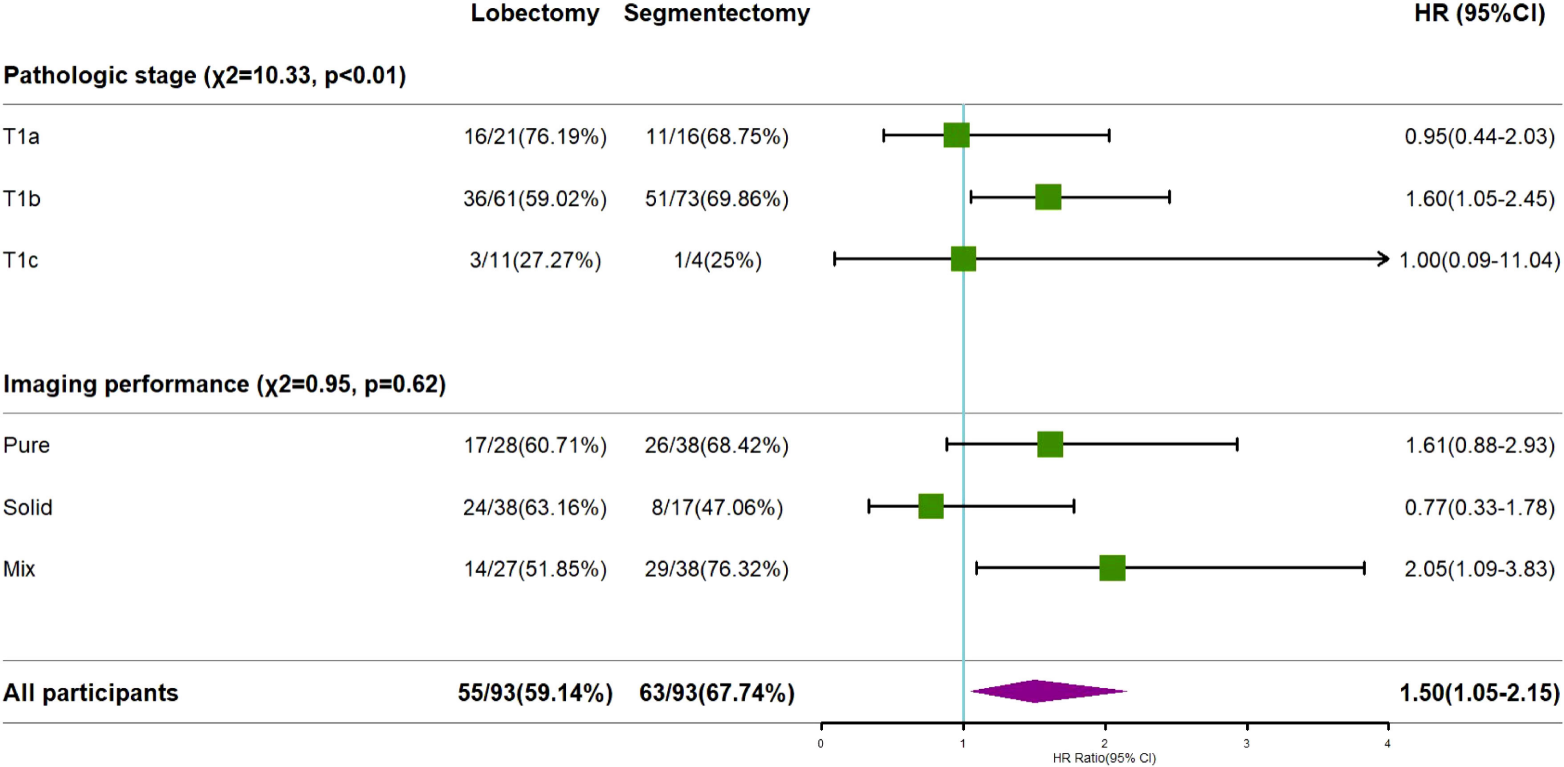
The effect of imaging findings or pathological stage on the recurrence of invasive lung adenocarcinoma.

Overall, the risk of recurrence was higher in the segmentectomy group than the lobectomy group, with an HR value of 1.5 (>1) and significant at the 5% level, which suggests that the treatment effect in the segmentectomy group was inferior to that in the lobectomy group, resulting in a 50% higher probability of recurrence. The pathological stage was also identified as a crucial risk factor for postoperative recurrence (χ²=10.33, p<0.01). Subgroup analysis results indicated no significant difference in treatment outcomes between T1a and T1c, while the risk of recurrence in the T1b segmentectomy treatment group was significantly higher than in the lobectomy group (HR=1.6>1, p<0.05). The imaging findings did not significantly impact the risk of postoperative recurrence (χ²=0.95, p=0.62>0.05). However, subgroup analysis results indicated no significant difference between the two treatments in the pure and solid adenocarcinoma groups. In contrast, the recurrence risk of the segmentectomy group was significantly higher than the Lobectomy group in the mixed group (HR value = 2.05, p< 0.05), suggesting the superior performance of the Lobectomy group (**Figure 6**).

## 4 Discussion

Surgical resection, especially lobectomy, is the current standard of care for stage I NSCLC. However, an estimated 30% of patients have surgically resectable tumors that are not clinically suitable for lobectomy (15). Segmentectomy has traditionally been considered a compromising procedure for patients with poor lung function, advanced age, severe comorbidities, or other reasons why lobectomy cannot be performed (16). Growing evidence supports the equivalence of segmentectomy and lobectomy for early-stage NSCLC. A meta-analysis (17) based on 28 studies of patients with stage I NSCLC showed that the prognosis was similar for segmentectomy and lobectomy when the tumor was less than 2 cm in diameter. Published results of the JCOG0802 study (18) demonstrated the efficacy of segmentectomy in small, peripheral NSCLC. Patients with stage IA NSCLC who underwent segmentectomy had a higher survival rate than those with lobectomy, despite a higher local recurrence rate. In the CALGB140503 trial (19), a large randomized study, sublobar resection was found to be non-inferior to lobectomy in terms of disease-free survival (primary endpoint) for patients with peripheral clinical stage T1aN0 (≤2 cm) NSCLC. Additionally, both procedures yielded comparable overall survival rates (secondary endpoint). However, few studies have investigated the applicability of pulmonary segmentectomy in patients with IAC pathology. Two studies (20) found that lobectomy did not provide better OS and RFS than segmentectomy, regardless of pathological subtype. Studies (21–23) that examined the equivalence of lobectomy to segmentectomy in patients with early-stage IAC showed that segmentectomy outcomes were similar to lobectomy only in older patients (≥65 years). Consequently, these findings cannot be extrapolated to patients outside this age range. The present study assessed patients aged 31 to 85 years and observed that segmentectomy and lobectomy demonstrated similar OS for early-stage patients with IAC pathology, consistent with the literature. However, the RFS of segmentectomy was significantly lower than that of lobectomy.

Interestingly, some studies (24, 25) recommend lobectomy for younger patients and segmentectomy for older patients, as the latter yields better perioperative outcomes without affecting long-term oncology outcomes. In our study, the age of the two groups of patients was similar, suggesting that the patient’s age may not be a determining factor in the selection of surgical procedures (26). This study compared the perioperative efficacy and safety of segmentectomy and lobectomy in patients with pathological IAC. Moreover, the intraoperative blood loss (P=0.014), postoperative catheter retention time (P=0.005), and postoperative hospital stay (P=0.002) in the segmentectomy group were significantly lower than in the segmentectomy group. In addition, the two groups exhibited no significant difference in the incidence of complications. Importantly, segmentectomy ensures a smaller scope of lung resection, preserving more lung tissue, which leads to better lung function and improved quality of life after surgery. However, segmentectomy resections require more detailed dissection of the hilar vessels and bronchial structures, making them more difficult and time-consuming than lobectomy (27–30). However, due to the lack of data on lung function and surgical duration, we could not conduct specific analyses on these factors in our study. Nevertheless, we observed an incidence of deep vein thrombosis (DVT) ranging between 7.5% and 10.8%. This occurrence was primarily attributed to intramuscular venous thrombosis, which we diagnosed using color ultrasound imaging of both lower limbs. Our standard treatment protocol involved administering an anticoagulant dose of 1mg/kg heparin and seeking consultation with a vascular surgeon.

Based on data from 186 patients with early IAC who underwent major lung resection, Cox univariate and multivariate regression analyses showed that postoperative CAR was an independent risk factor for RFS in patients with IAC, with a postoperative CAR >0.25 associated with a high likelihood of recurrence. Surgical trauma can cause a systemic inflammatory response, including IL-1 (interleukin-1), IL-6 (interleukin-6), and TNF-alpha (tumor necrosis factor-alpha). IL-6 induces the activation of STAT3 and NF-*κ*B by CRP and specific acute phase response proteins, preventing apoptosis and thus promoting tumor cell proliferation (31). It is widely acknowledged that plasma proteins are synthesized in the liver and secreted into the circulation, including albumin, c-reactive protein (CRP), amyloid A, antitrypsin-alpha1, and acidic glycoprotein-alpha1, which are recognized markers of systemic inflammation in the acute phase (32). Recently, CAR has been identified as a new prognostic score. Preoperative CAR was an independent prognostic factor for OS and cancer-specific survival (CS) in elderly patients with stage I NSCLC undergoing major lung resection (8). The impact of preoperative CAR on the prognosis of the patients included in this study was not further analyzed due to the wide age range of the patients and the fact that most had an underlying disease and no significant abnormalities on preoperative tests. The severity of the postoperative inflammatory response can be influenced by the difference in the extent of surgical trauma caused by a segmentectomy and a lobectomy. Pulmonary segmentectomy greatly reduces the damage to the patient’s nerves, muscles, and blood vessels while maintaining the quality of the procedure. This study showed that postoperative leukocytes, neutrophils, SII, and NLR were significantly different in a controlled study of segmentectomy resection versus lobectomy, although they were not independent risk factors for RFS (*P*<0.05). The postoperative leukocytes, neutrophils, SII, and NLR, were lower in the segmentectomy compared to the lobectomy group, indicating that segmentectomy yields better outcomes for patients with early IAC in reducing the postoperative inflammatory response than lobectomy.

Although there were no statistically significant differences in the distribution of imaging classification and pathological stage among the study groups, we conducted subgroup analyses to explore potential confounding factors related to imaging classification and pathological classification. Based on the results of these subgroup analyses, we found that there was no significant difference in recurrence rates between the two surgical methods for T1a patients. However, T1b patients who underwent segmentectomy exhibited a higher risk of recurrence. These findings suggest that segmentectomy may be beneficial for T1a patients, particularly those with pGGO and Solid subtypes according to subgroup analysis of Imaging performance. In contrast, lobectomy may reduce the risk of recurrence in mGGO subtype patients. However, it does not mean that segmentectomy for pGGO and Solid patients in the T1a and Imaging performance subtypes will necessarily yield high benefits, but segmentectomy is a good surgical procedure to be considered for such patients.

The limitations of this study should be acknowledged. First, this study was a single-center retrospective analysis, and although a 1:1 propensity score matching was used, there were still shortcomings, such as selection bias and a relatively small sample size. Moreover, the intercept values in this study were based on post-matching study data only, and the exact conclusions have yet to be verified in a large, multicenter, prospective study. Besides, the observation period was not long enough. A longer follow-up time is warranted to analyze the relationship between surgical approach, tumor recurrence, and overall survival. Finally, data on postoperative pulmonary function and time to surgery were limited. Accordingly, further studies should focus on the differences in pulmonary function and time to surgery.

## 5 Conclusion

In summary, thoracoscopic segmentectomy is a safe surgical approach. Although its surgical outcomes are superior to lobectomy yielding a similar OS, caution should be taken when choosing this surgical approach for patients with IAC, given its high recurrence rate. More studies are warranted to refine the selection criteria for lung segmentectomy and optimize patient outcomes. Postoperative CAR is a simple, rapid, and inexpensive prognostic factor that can be used as a prognostic indicator for early IAC patients. Given the high risk of recurrence, more emphasis should be placed on patients with postoperative CAR >0.25.

## Data availability statement

The raw data supporting the conclusions of this article will be made available by the authors, without undue reservation.

## Ethics statement

The studies involving humans were approved by the ethical review committee of Jiaxing First Hospital. The studies were conducted in accordance with the local legislation and institutional requirements. The participants provided their written informed consent to participate in this study. Written informed consent was obtained from the individual(s) for the publication of any potentially identifiable images or data included in this article.

## Author contributions

CD: Conducted the study. Collected, analyzed, and interpreted the data. Wrote the manuscript. QJ: Designed the study, and interpreted the data, and edited the manuscript. ZW: Planned the project. Reviewed the manuscript. YZ: Interpreted the data. YH: Edited the manuscript, reviewed the manuscript. JW: Edited the manuscript. DW: Reviewed the manuscript. All authors read and approved the final manuscript.
